# Health-related quality of life in ICU survivors—10 years later

**DOI:** 10.1038/s41598-021-94637-z

**Published:** 2021-07-26

**Authors:** José G. M. Hofhuis, Augustinus J. P. Schrijvers, Tjard Schermer, Peter E. Spronk

**Affiliations:** 1 Department of Intensive Care, Gelre Hospital, Albert Schweitzerlaan 31, 7334 DZ Apeldoorn, The Netherlands; 2 Department of Epidemiology, Gelre Hospital, Albert Schweitzerlaan 31, 7334 DZ Apeldoorn, The Netherlands; 3 Julius Center for Health Sciences and Primary Care, University Medical Center, Heidelberglaan 100, 3584 CX Utrecht, The Netherlands; 4 Department of Intensive Care, Academic Medical Center, Meibergdreef 9, 1105 AZ Amsterdam, The Netherlands; 5 Radboud Institute for Health Sciences, Radboud University Medical Center, Geert Grooteplein 21, 6525 EZ Nijmegen, The Netherlands

**Keywords:** Health care, Medical research

## Abstract

Many Intensive Care (ICU) survivors experience long lasting impairments in physical and psychological health as well as social functioning. The objective of our study was to evaluate these effects up to 10 years after ICU discharge. We performed a long-term prospective cohort study in patients admitted for longer than 48 h in a medical-surgical ICU. We evaluated health-related quality of life (HRQOL) before ICU admission using the Short-form-36 (SF-36), at ICU discharge, at hospital discharge and at 1, 2, 5 and 10 years follow up (all by patients). Changes in HRQOL were assessed based on linear mixed modeling. We included a total of 749 patients (from 2000 to 2008). During 10 years 475 (63.4%) patients had died, 125 (16.7%) patients were lost to follow up and 149 (19.9%) patients could be evaluated. The mean scores of four HRQOL dimensions (i.e., physical functioning (p < 0.001; mean 54, SD 32, effect size 0.77, 95% CI [0.54–1.0]), role-physical (p < 0.001; mean 44, SD 47, effect size 0.65, 95% CI [0.41–0.68] general health (p < 0.001; mean 52, SD 27, effect size 0.48; 95% CI 0.25–0.71) and social functioning (p < 0.001; mean 72, SD 32, effect size 0.41, 95% CI [0.19–0.64]) were still lower 10 years after ICU discharge compared with pre-admission levels (n = 149) and with an age reference population. Almost all SF-36 dimensions changed significantly over time from ICU discharge up to 10 years after ICU discharge. Over the 10 year follow up physical functioning of medical-surgical ICU survivors remains impaired compared with their pre-admission values and an age reference population. However, effect sizes showed no significant differences suggesting that surviving patients largely regained their age-specific HRQOL at 10 years.

## Introduction

There are a growing number of survivors of critical illness due to the aging population and with lower numbers of short term mortality in the intensive care unit (ICU)^1,2^. Nevertheless, the long-term propensity to die remains higher than age and sex matched healthy controls^3,4^. Many ICU survivors experience a reduced physical and psychological health as well as impaired social functioning after ICU discharge. These factors seem inter-related, because functional disability was found associated with a reduction in health-related quality of life^5^. However, studies on HRQOL in those patients are hampered by several drawbacks. Health-related quality of life (HRQOL) after ICU stay is frequently evaluated at 6 months after the critical illness which may result in recall bias^5–7^. Also, response shift may play a role, i.e. the capacity of a person to variate their own balance between attained goals and capacities. In that setting, measuring the burden of critical illness is difficult due to the different individual health states before and after intensive care admission^8,9^. Furthermore, one may argue that all studies of ICU survivors are biased since these are, in fact, survivorship cohorts where the younger, less complex patients with more biological reserve will survive and bias long-term follow-up. Interestingly, a study of our group in octogenarians showed a good recovery of HRQOL after 6 months in patients surviving critical illness compared with pre-admission^10^. Indeed, it is important to recognize that patients may be on different post-ICU outcome trajectories and by combining all survivors into one group, these subtleties in outcome may be lost^11^. To accommodate part of these problems, as shown in our previous 5 years follow-up study^12^, we chose to use a Dutch reference general population^13^. Despite these drawbacks, HRQOL remains the most commonly reported long-term outcome after critical illness. Most of these studies performed in general ICU patients after ICU discharge did not exceed a follow up period of 2–5 years, nor did they evaluate the HRQOL before the patients became seriously ill and were admitted to the ICU^14–16^. However, studies in different patient groups (i.e. health subjects and patients with cardiac or gastrointestinal disease), did investigate a 10 year follow up period to analyze the long-term course of HRQOL^17–19^.

Therefore, we hypothesized that 10 years after ICU discharge, perceived HRQOL of survivors is comparable to their pre-admission level and an age reference population. In the present study, we aimed to assess the impact of ICU stay and change of HRQOL up to 10 years. In addition, we compared the HRQOL of the surviving patients with an age reference population.

## Methods

Between September 2000 until January 2008 we performed a long-term prospective cohort study in a 10 bed closed-format (intensivist led) mixed medical-surgical ICU in a 654-bed university-affiliated teaching hospital in Apeldoorn, The Netherlands. This is the primary analysis of the data acquired at 10 years after ICU discharge after finishing the analyses of this cohort after 5 years follow-up^12^. The findings were compared with previously obtained data in the same cohort that were also included in previous studies by our group^12,20–22^*.* The patient population cared for comprises adult medical patients (all diagnoses) and adult surgical patients except neurosurgery, cardiac surgery, and transplant surgery. All admissions were screened for study participation (Additional file-1). The hospital’s ethics committee approved the study to apply an oral informed consent at the beginning of this long-running study. A patient’s consent was confirmed and recorded in writing in the patient's medical record. Additionally, when patients were contacted after 5 and 10 years they were orally asked again to confirm their previously given informed consent to participate in the study. We made every effort to accomplish the highest response rate possible, by contacting the ICU survivors again via telephone and mail, and all available data (including the data of the non-survivors) were used in the linear mixed model. All research was performed in accordance with relevant guidelines/regulations. The STROBE Statement checklist for observational studies was used when writing this manuscript^23^.

We evaluated HRQOL before admission (proxies), ICU discharge, hospital discharge and 1, 2, 5 and 10 years after ICU discharge. We initially only included patients with an ICU stay > 48 h, because we aimed to evaluate the sickest patients, hypothesizing that the impact of ICU stay on HRQOL would be most prominent in those cases. We have shown in a previous study^24^ that there is no added value of including ICU patients admitted for 48 h or less. Furthermore, patients with no close proxy, re-admission on the ICU, an impaired level of self-awareness or without the ability to communicate adequately at any time during the study, cognitive impairment, or transferred to another hospital were excluded^12^. Patients’ demographic data and severity of illness (Acute Physiology and Chronic Health Evaluation)^25^ were also collected.

### Health-related quality of life measurement

The SF-36 (version 1)^26^, a widely used standardized generic health status questionnaire, was used to measure HRQOL. This study is an extension of our first study up to five years and part of an ongoing project. Detailed information about the methods and procedures are described elsewhere^12^. As most of the ICU patients are not able to complete a questionnaire at the time of admission, proxies have to be used frequently as a surrogate approach. The use of proxies to assess the patient’s health-related quality of life was validated in earlier studies by our research group using the SF-36^27^ and the Academic Medical Center Linear Disability score measuring physical reserve^28^. Importantly, proxies had to be in close contact with the patient on a regular basis, were asked to answer on behalf of the patient, and mark the statement that best described the patient’s state of health in the last four weeks prior to the admission. Procedures used to assess the SF-36 are described in the 5 years follow up study of our group^12^. To evaluate the differences between patient’s reported HRQOL with those of age controls, we compared HRQOL before ICU admission and 10 years after ICU discharge with those of an age reference Dutch population^13^ and used the first question of the SF-36 as a measure of the perceived overall health state. This is the single-item question pertaining to general health status: “In general would you say your health is excellent, very good, good, fair or poor?” No statistical power calculation was conducted prior to the study. The sample size was based on the inclusion of as many eligible and consenting patients during the accrual period of the study (September 2000 until January 2008).

### Statistical analysis

As we aimed to assess how patients improve after ICU discharge, we chose to analyze changes over time from ICU discharge using a linear mixed model for each dimension of the SF-36 using the pre-ICU score as a covariate^29^. The main advantage of such a model is that each measurement of each subject is used, regardless of time of drop-out (like mortality). These models are less biased than complete-case analyses, as also the ‘worse’ patients who eventually drop out of the study are included as much as possible in the estimations of change over time. Including also patients who drop out will have a negative impact on the estimates of improvement over time. The improvement from ICU-discharge is estimated using data obtained directly from patients, the proxy assessment at baseline is used only to correct for differences in pre-ICU HRQOL between patients. We made the following technical choices in the linear mixed model: a random intercept model, in which patients were included as a random effect (i.e. allowed to deviate from the common intercept); fixed effects included time, pre-ICU SF-36 score, Acute Physiology and Chronic Health Evaluation, age and gender; and the final estimation method was full maximum likelihood. Variables that did not significantly contribute to the model by consecutively excluding variables with the highest p-value from the model until only variables with p < 0.20 remained, were excluded using a backward exclusion approach. The assumption of normality of the residuals was assessed by a Q–Q plot. Estimates of domain scores at different time points are presented with 95% confidence intervals. To present the simplest possible model, we used the Bayesian Information Criterion to determine whether random slopes needed to be included in the model. We chose to report the models with random slopes for time (i.e. a different slope/trajectory for each patient), as these were significantly better than models without random slopes in all domains. Time was added as a quadratic variable; all other continuous variables are used without transformations. As we had minor missing data of the included variables and the outcome data and since linear mixed models provide unbiased estimates in the presence of missing outcomes (that are missing completely at random), we decided (in consultation with a statistician) that it was neither necessary nor appropriate to impute missing data.

For the comparison of pre-admission versus 10 years follow up SF-36 scores, we could not use the linear mixed model, as the pre-admission score was included in that model as a covariate. Therefore, we performed one-way analyses of covariance (i.e. a general linear model) with Bonferroni correction^29^ to detect differences in the SF-36 scores at admission between survivors and non-survivors and to asses changes between pre-ICU and 10 years after ICU discharge (repeated measures analyses of covariance). Statistical adjustment was made for age, sex and Acute Physiology and Chronic Health Evaluation^30^ by including these variables as covariates. No variables were analyzed as effect modifiers.

SF-36 dimensions of survivors were compared with normative data from the age group 60–70 years from the Dutch reference population^13^ using the one sample T test. The significance level was adjusted by Bonferroni correction according to the number of related tests conducted. To examine the relative magnitude of changes over time and between groups, effect sizes were used based on the mean change found in a variable divided by the baseline standard deviation.

Effect sizes estimate whether particular changes/differences in health status are relevant, helping to interpret mean differences. Following Cohen, effect sizes of ≥ 0.20, ≥ 0.50, and > 0.80 were considered small, medium, and large changes, respectively^31^. To illustrate the course of health-related quality of life over time, we plotted raw (uncorrected) data**.** Groups were defined on the length of follow up (i.e. ranging from only pre-ICU to 10 years after discharge).

X^2^ tests were used to assess the demographic differences between ICU survivors and ICU non-survivors. Data were analyzed using the Statistical Package for the Social Sciences (SPSS Inc, Chicago IL, USA, version 17). All analyses were tested two-tailed. All data are expressed as mean ± SD where appropriate unless otherwise indicated.

### Ethics approval

The hospital’s ethics committee of Gelre Hospital Apeldoorn, the Netherlands approved the study.

## Results

During the study period, 3775 patients were screened for study participation. We included a total of 749 patients (20%) (Additional file-1). Out of those patients 61% were men and 39% women. Baseline SF-36 scores were obtained from all patients who were evaluated in the final analysis. In addition to ICU discharge and 3,6,12 months after ICU discharge (Table 1), HRQOL was measured at 5 years (n = 234) and 10 years after discharge (n = 149). At 10 years, a total of 125 patients (16.7%) were lost to follow up (mentally impairment, dementia, long-term delirium (n = 75), no contact possible (n = 13), or due to a transfer to another hospital (n = 37). Ten years mortality of the total group was 63.4% (n = 475). The baseline demographic and clinical characteristics of the patients lost to follow-up did not differ significantly from the group analyzed in the study except for some types of admission and diagnostic groups (Additional file-2). The demographic and clinical characteristics of all patients are shown in Table 2.

**Table 1:** Health-related quality of life from ICU admission to 10 years after ICU discharge.

	Survivors N =	Physical component	Mental component	Physical functioning	Role-physical	Bodily pain	General health	Vitality	Social functioning	Role-emotional	Mental health
Pre-morbid	749	41 ± 13	48 ± 10	59 ± 34	51 ± 48	79 ± 27	51 ± 30	53 ± 24	72 ± 25	74 ± 41	67 ± 17
ICU discharge	547	27 ± 6	46 ± 9	6 ± 13	14 ± 32	76 ± 25	31 ± 19	33 ± 16	52 ± 23	62 ± 44	57 ± 12
Hospital discharge	446	32 ± 9	48 ± 10	30 ± 26	19 ± 35	82 ± 24	38 ± 26	45 ± 18	60 ± 27	67 ± 46	63 ± 13
3 months	412	37 ± 11	50 ± 11	49 ± 32	29 ± 41	81 ± 24	44 ± 26	56 ± 22	69 ± 27	72 ± 42	68 ± 16
6 months	398	39 ± 11	50 ± 11	54 ± 32	39 ± 45	83 ± 23	46 ± 24	58 ± 22	73 ± 25	76 ± 41	69 ± 20
1 year	378	40 ± 12	51 ± 10	59 ± 31	49 ± 46	82 ± 22	47 ± 26	59 ± 21	71 ± 25	82 ± 37	69 ± 19
2 years	301	40 ± 12	51 ± 10	54 ± 31	49 ± 46	80 ± 24	49 ± 25	61 ± 19	73 ± 25	77 ± 41	69 ± 14
5 years	234	39 ± 12	52 ± 10	57 ± 32	43 ± 46	77 ± 26	49 ± 26	61 ± 21	75 ± 26	82 ± 42	71 ± 15
10 years	149	38 ± 13	53 ± 9	54 ± 32	44 ± 47	77 ± 28	52 ± 27	63 ± 20	72 ± 32	81 ± 39	74 ± 14
Dutch normal Population (61–70 years)		49 ± 9	52 ± 10	72 ± 26	67 ± 41	71 ± 25	62 ± 20	68 ± 20	82 ± 25	81 ± 35	77 ± 18

Values indicate mean ± SD. SF-36 dimension scores are 0–100 scores. Physical Component Score and Mental component scores are converted to mean 50 (SD 10).

**Table 2:** Demographic and clinical characteristics of patients included in the study.

Median (IQR)	Total group	5 year survivors after ICU discharge	10 year survivors after ICU discharge	Non survivors up to 10 Years	Differences survivors vs non-survivors 10 years
**N = **	749	234	149	475	P value
Age total group (years)	71 (62–77)	66 (55–74)	64 (54–70)	73 (66–79)	< 0.001
Sex: Male N (%)	457 (61)	133 (57)	85 (57)	295 (62)	< 0.001
Female N (%)	292 (39)	101 (43)	65 (43)	180 (38)	< 0.001
Acute physiology and chronic health score (points)	19 (14–23)	18 (13–22)	17 (13–21)	19 (16–24)	< 0.001
ICU length of stay (days)	8 (5–15)	7 (5–15)	7 (5–15)	8 (5–16)	0.509
Hospital length of stay (days)	23 (13–39.5)	26 (16–42.2)	26 (16–41)	22 (13–41)	0.057
Ventilation days	6 (3–12)	5 (2–10)	5 (2–10)	6 (3–13)	0.162
**Diagnostic groups N (%)**					
Cardiovascular pathology	184 (24.6)	63 (27)	41 (27)	115 (24)	< 0.001
Respiratory pathology	244 (32.6)	66 (28)	38 (25)	158 (33)	< 0.001
Gastrointestinal pathology	259 (34.6)	86 (37)	55 (37)	174 (37)	0.609
Neurological pathology	30 (4.0)	6 (3)	6 (4)	16 (3)	0.033
Trauma	23 (3.1)	10 (4)	8 (5)	6 (1)	0.593
Others	9 (1.2)	3 (1)	2 (1)	6 (1)	0.157
**Type of admission N (%)**					
Non-surgical	415 (55.4)	113 (48)	70 (47)	259 (55)	< 0.001
Emergency surgical	257 (34.3)	95 (40.5)	65 (43)	156 (33)	< 0.001
Elective surgical	77 (10.3)	26 (11.5)	15 (10)	60 (13)	< 0.001
**Type of proxy N (%)**					
Spouse	523 (69.8)	147 (63.0)	70 (47)	298 (62)	< 0.001
Children	213 (28.4)	84 (36.0)	79 (53)	170 (36)	< 0.001
Brother/Sister	13 (1.7)	3 (1.0)	0	7(2)	-

Elective surgical: ICU admission was planned within a 24 h period before surgery, Emergency surgical: unplanned surgery, Non-surgical: all other admissions.

Values indicate medians and interquartile range (P_25_-P_75_) unless stated otherwise Only important diagnostic groups were added.

### Changes over time in patients up to 10 years after ICU discharge

**Figure 1:**
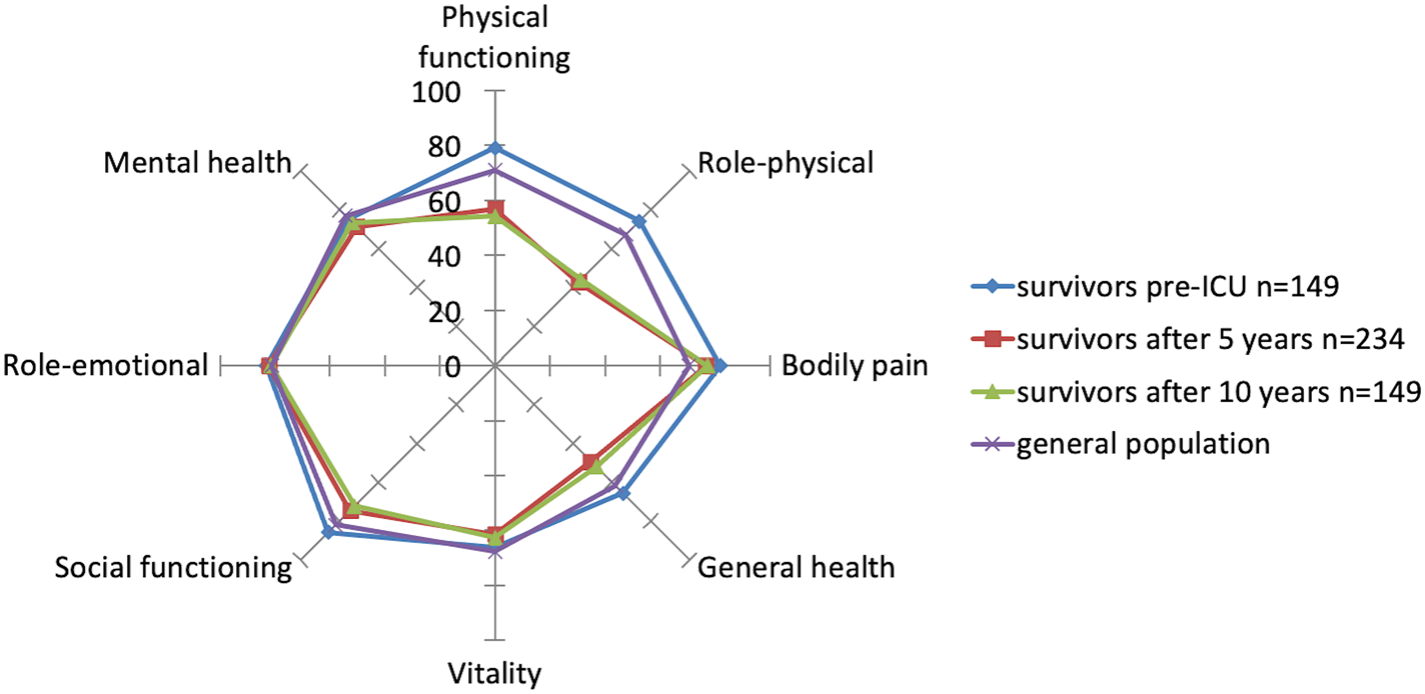
Comparisons of mean Short-form 36 scores of survivors before ICU admission, 5 and 10 years after ICU. Values in the different domains are all normalized to a scale of 0–100.

**Figure 2:**
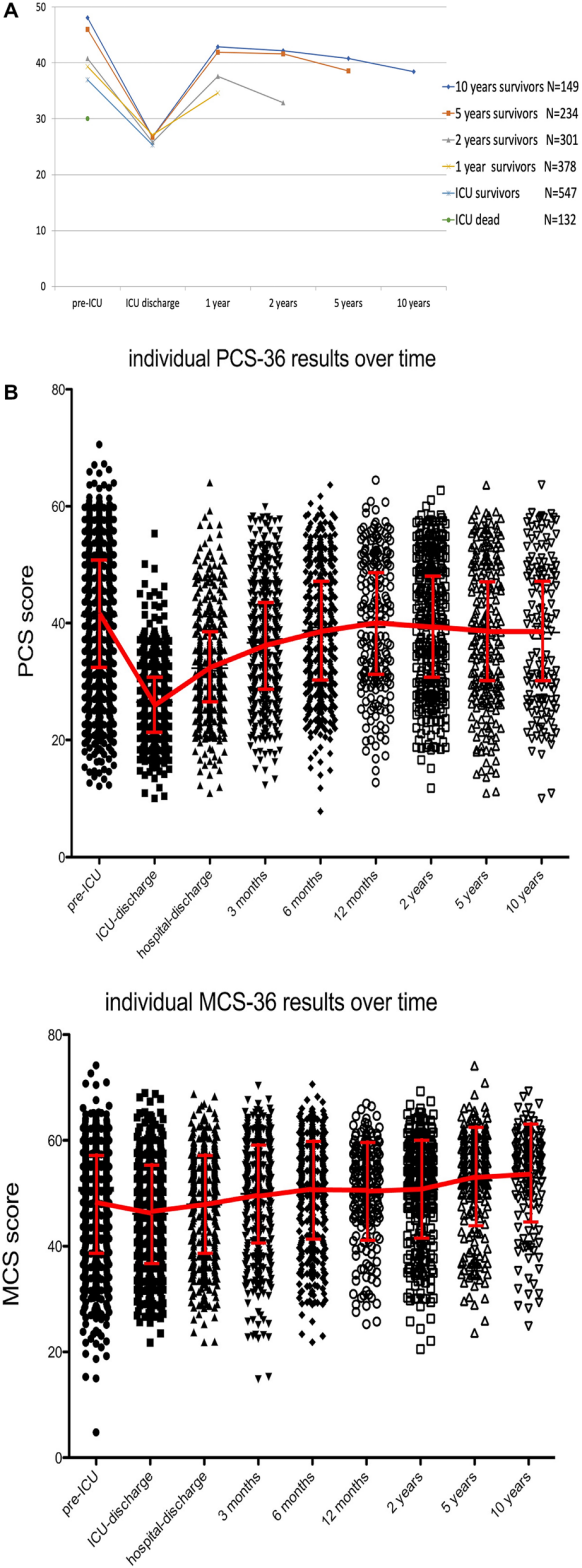
Panel (**A**) Course of mean physical component score over time. with different survival time (raw uncorrected data). Panel (**B**) Individual physical component score Short form F-36 and mental component score Short Form-36 results over time; Red lines indicate mean values.

The linear mixed model^29^ showed that almost all SF-36 dimensions changed significantly over time from ICU discharge up to 10 years after ICU discharge, except for bodily pain (Table 3, Fig. 1). Pre-ICU HRQOL score was a significant predictor of change in contrast to the Acute Physiology and Chronic Health Evaluation^30^. At ICU discharge the HRQOL scores were lowest for physical functioning, role-physical, general health and vitality dimensions. Bodily pain had the highest score. The course of HRQOL over time and individual time assessments are illustrated in Fig. 2 (panel A,B) using uncorrected values, i.e. not derived from the linear model.

**Table 3:** Estimates of change over time from ICU discharge*.

N = 149 Short-form 36 dimensions	ICU-discharge (Intercept)	95% CI	Change per month	95% CI	Pre-ICU score #	95% CI	Interaction time	95% CI
Physical component	25.58	22.18 to 28.96	0.17	0.13 to 0.21	0.26	0.21 to 0.31	− .001	.002- to .001
Mental component	38.76	34.84 to 42.68	0.11	0.08 to 0.15	0.21	0.14 to 0.27	− .001	− .001 to − .000
Physical functioning	16.68	8.78 to 24.59	0.41	0.30 to 0.51	0.43	0.36 to 0.48	− .003	− .004 to − .002
Role-physical	24.76	15.75 to 33.78	0.63	0.46 to 0.80	0.16	0.10 to 0.21	− .004	− .006 to− .003
General Health	30.28	23.95 to 36.62	0.14	0.04 to 0.23	0.22	0.17 to 0.28	− .001	− .001 to− .000
Mental Health	47.52	42.04 to 52.99	0.09	0.04 to 0.15	0.28	0.20 to 0.34	− .000	− .00 to − 1.52
Bodily pain	80.61	74.04 to 87.17	-0.12	− .22 to − .035	0.37	− .019 to .095	.000	− .000 to .001
Role-emotional	65.24	56.16 to 75.31	0.28	0.11 to 0.16	0.09	0.03 to 0.16	− .001	− .003 to − .000
Social functioning	45.20	37.58 to 52.82	0.26	0.17 to 0.35	0.31	0.24 to 0.38	− .002	− .00- to − .001
Vitality	39.53	33.64 to 45.42	0.27	0.19 to 45.4	0.23	0.17 to 0.29	− .001	− .00- to -.001

*linear mixed model with random intercept and random slope (for time).

^#^Pre-ICU estimate: change in discharge-ICU score (intercept) for one point higher pre-ICU score.

*ICU* intensive care unit, *CI*  confidence interval.

### Comparison of survivors after 10 years with reference general population

Surprisingly, of the patients who survived after 10 years, the pre-admission HRQOL was significantly better in two dimensions with small effect sizes i.e. physical functioning (p < 0.001; mean 79, SD 26, effect size 0.28, 95%CI [0.12–0.45]) and bodily pain (p < 0.001; mean 82, SD 26, effect size 0.45, 95%CI [0.29–0.61]), compared with an age reference population. At 10 years, the HRQOL was significantly lower in four dimensions with medium effect sizes in the physical functioning (p < 0.001; mean 54 SD 32, effect size 0.54, 95% CI [0.38–0.70]), role limitation due to physical problems (p < 0.001; mean 44, SD 47, effect size 0.50, 95% CI [0.38–0.66]), the general health with small effect size (p < 0.001; mean 52, SD 27, effect size 0.36, 95% CI [0.20–0.52]) and the social functioning (p < 0.001; mean 72, SD 32, effect size 0.30, 95% CI [0.14–0.46]). Effect sizes in all other dimensions were small (< 0.50). The significant difference in the bodily pain dimension was based on a higher 10-year score (mean 77.1) compared with the general population (mean 70.5) (Table 4).

**Table 4:** Health-related quality of life in surviving patients and comparison with Dutch general population.

Short form-36 dimen-sions Mean ± SD	Pre-ICU of all patients	Pre-ICU of all 10 years survivors	10 years after discharge	General population **(**age group 61–70)	Differences between pre-ICU and 10 years after ICU discharge (N = 149)#		Differences between 5 and 10 years after ICU discharge (N = 149) #	Differences between pre-ICU survivors (N = 149) and pre-ICU non survivors (N = 475) †		Differences between 10 years after ICU discharge with general population **		Differences between Pre-ICU survivors with general population **	
N =	749	149	149		Wilks' Lambda	Effect sizes	Wilks ’ effect Lambda sizes	p value	Effect sizes	p value	Effect sizes	p value	Effect sizes
Physical-component	41 ± 13	48 ± 11	38 ± 13	–	–	–	0.005* 0.19	< 0.001	0.74	–	–	–	–
Mental-component	48 ± 10	52 ± 9	53 ± 9	–	–	–	0.326 0.09	< 0.001*	0.44	–	–	–	–
Physical-functioning	59 ± 34	79 ± 26	54 ± 32	72 ± 26	< 0.001*	0.77	< 0.001* 0.32	< 0.001*	0.81	< 0.001*	0.54	0.001*	0.28
Role-physical	51 ± 48	74 ± 42	44 ± 47	67 ± 41	< 0.001*	0.65	0.458 0.05	< 0.001*	0.71	< 0.001*	0.50	< 0.046	0.17
Bodily pain	79 ± 27	82 ± 26	77 ± 28	71 ± 25	0.119	0.17	0.371 0.08	0.041	0.19	0.002*	0.24	< 0.001*	0.45
General-health	50 ± 30	66 ± 28	52 ± 27	62 ± 20	< 0.001*	0.48	0.335 0.08	< 0.001*	0.70	< 0.001*	0.36	0.088	0.14
Vitality	53 ± 24	66 ± 25	63 ± 20	68 ± 20	0.239	0.13	0.108 0.13	< 0.001*	0.67	0.002*	0.25	0.453	0.06
Social-functioning	72 ± 25	86 ± 20	72 ± 32	82 ± 25	< 0.001*	0.41	0.003* 0.24	< 0.001*	0.71	< 0.001*	0.30	0.022	0.19
Role-emotional	74 ± 41	86 ± 34	81 ± 39	81 ± 35	0.242	0.12	0.118 0.15	< 0.001*	0.43	0.917	0.007	0.071	0.13
Mental-Health	67 ± 17	75 ± 16	74 ± 14	77 ± 18	0.281	0.11	0.860 0.01	< 0.001*	0.63	0.003*	0.25	0.193	0.11

Effect size: ≥ 0.20 small, ≥ 0.50 medium, > 0.80 large; Values indicate mean ± SD.

^**†**^ Univariate Analysis of Variance with Bonferroni correction p < 0.05 significant # GLM repeated measures with Bonferroni correction p < 0.05 significant.

** One sample T test. * P value significant after Bonferroni correction (p 0.05/10 = p = 0.005 = significant). SF-36 dimension scores are 0–100 scores.

Population scores on Physical component score and Mental component scores have been standardized on a population mean of 50 with a SD of 10.

### Development of health-related quality of life over time in survivors and non-survivors

The mean scores of four dimensions, i.e. physical functioning (p < 0.001; mean 54, SD 32, effect size 0.77, 95% CI [0.54–1.0]), role-physical (p < 0.001; mean 44,SD 47, effect size 0.65, 95% CI [0.41–0.68]), general health (p < 0.001, mean 52, SD 27, effect size 0.48, 95% CI [0.25–0.71]) and social functioning (p < 0.001, mean 72, SD 32, effect size 0.41, 95% CI [0.19–0.64]), with medium- small effect sizes were still lower 10 years after ICU discharges compared with their pre-admission levels (n = 149) (Table 4). Obtained values of HRQOL domains are shown in Table 1. The physical functioning dimension (p < 0.001; mean 54, SD 32, effect size 0.32, 95% CI [0.08–0.54]) and the social functioning (p = 0.003; mean 72, SD 32, effect size 0.24, 95% CI [0.007–0.46]) of HRQOL was significant lower with small effect sizes at 10 years compared with 5 years (Table 4). Pre-admission scores of non-survivors were significantly lower in all dimensions compared with the 10-year survivors (all p < 0.001), except bodily pain; (p = 0.041; Additional file-3).

## Discussion

This is the first prospective cohort study evaluating long-term effects of ICU stay on health-related quality of life at different time points including the pre-ICU status over a prolonged period up to 10 years after ICU discharge. Improvement was strongest at 10 years in the domains physical health and role-physical, and intermediate in vitality and social functioning domains. Nevertheless, HRQOL is still significantly decreased in three dimensions of the SF-36 with medium effect sizes in the physical functioning and role limitation due to physical domains, and with small effect size in the general health domain compared with an age reference population. Studies that measured follow up in a general group of ICU patients for 5 years or longer are limited^15,16,32–36^. Herridge found that relatively young patients who survived had persistent exercise limitations and a reduced physical quality of life 5 years after their critical illness^16^. The outcome of ICU treatment is mostly reported as mortality or report on a specific diagnostic group^37,38^. Ten years mortality after ICU discharge of our study was 63% and somewhat higher than found by Stricker after 9 years^33^. This may be due to the fact that Stricker included patients who were admitted longer than 24 h while we included only patients with an ICU stay longer than 48 h and therefore those patients were possibly sicker with a higher probability of death.

We compared our study findings with an age reference population and more importantly we compared the HRQOL at 10 years with reported values before ICU admission. Baseline assessment (assessed on ICU admission) is important when investigating the impact of critical illness^39^. As more patients are surviving critical illness, assessing long-term outcomes becomes increasingly important^40^. There is no consensus regarding the follow up time of HRQOL studies. In this study we were interested in long-term effects, since one could also argue that recovery would again show a declining slope after an initial plateau effect^41^.

In the 10 year follow up period of our study, patients may have developed other health problems not related to the ICU reason of admission and it may be debatable whether functional outcome questionnaires can still yield useful and relevant information^39^. However, the burden that arises after ICU treatment can be strong, including long-term physical, functional and cognitive impairments^42,43^. To gain insight in our patients’ trajectories, as well as for the development of interventions after ICU discharge and in the home environment to improve HRQOL, it seems important to be informed on the physical and psychological changes of large cohorts of patients who survived critical illness^44^. General studies showed, as in our study, that psychological HRQOL is less affected than physical in critical care survivors (when measured with the SF-36) and therefore interventions within 1 year after ICU discharge to improve physical health might be more successful. However, there is increasing awareness on the psychological sequelae of ICU admission as well^44^. Factors that could be the reason for a poor HRQOL after ICU, such as age, prolonged ICU or hospital stay or long mechanical ventilation are not per se indicators of reduction afterwards^45,46^. Physical impairments after major trauma seems more concrete to influence long-term HRQOL than cognitive impairments, sleep-disturbances and post-traumatic stress disorder^46–49^. Furthermore, studies showed that ICU patients have more chronic conditions during the year before ICU admission compared with a population based control group^49,50^, and a five times higher odds on developing one or more chronic conditions compared with the control group during the year after admission^50^. To our knowledge it is unknown if long-term ICU survivors after 5–10 years have a higher risk of chronic diseases. Follow-up care after ICU may focus on the identification and treatment of the new developed chronic conditions^50^. These factors could be addressed in future research if we are to optimize long-term outcomes after critical illness.

In the past 1–2 decades critical care management has changed and an increasing proportion of patients survive the acute episode. However, those patients stayed longer in the ICU and in the hospital, with serious and lasting physical, cognitive and psychological problems and a greater dependency and health care utilization following discharge from hospital, demonstrating a substantial impact on health- related quality of life^16,35,51^. The path of recovery of those patients has led to the development of critical care follow up clinics, and rehabilitation after critical care. In the future we think ICU follow up clinics can help to identify patient-specific morbidity and arrange suitable post-ICU management to improve long-term outcomes.

Surprisingly, of the patients in our study who survived after 10 years, the pre-admission scores were significantly better in the physical functioning domain compared with an age reference population. The effect sizes, according to Cohen’s effect^31^, were small, suggesting that the effect of this finding may not be clinically significant. An important problem of long-term follow up is that more patients will be lost to follow up. However, in our study we made every effort to accomplish the highest response rate possible and all available data (including the data of the non-survivors) were used in the linear mixed model.

As this study is an extension of our previous 5 years study^12^, we chose to use a Dutch normal population as a reference population^13^ again to compare with the data of the 10 years survivors. Translation, validation and norming of the Dutch language version of the SF-36 health questionnaire have been evaluated in 1998 in community and chronic disease populations. Some studies chose to compare with data with other international groups or meta-analysis^52^. However, we think this approach could also be hampered due to differences in country populations and staffing differences.

### Strengths and limitations

Strengths of our study are that we repeatedly measured changes from ICU discharge to 10 years thereafter by the same observer (JH). Assessment of HRQOL as in our study is, ideally, conducted in a longitudinal design with multiple measurements over time^53^. Furthermore, HRQOL should be measured in each patient before and after ICU admission, because our main interest is the change in perceived health.

The measurement of pre-admission quality of life does provide an estimate of a patient’s physiologic and mental reserve, and may therefore be a significant determinant of short-term and long-term prognosis for ICU patients. Indeed, in line with what we showed in a previous study^24^, pre-admission quality of life measurement could potentially contribute to making decisions and optimal post-ICU patient management^54^.

Several limitations to our study should also be mentioned. First, in the period of 10 years between ICU discharge and study evaluation, HRQOL could have been influenced by other inter-current disease processes not related to the original ICU-stay. As such, the relationship between the patient’s reported HRQOL and the original ICU-admission may seem far-fetched. Nevertheless, we think it is interesting to report the actual situation of the perceived HRQOL in patients who survived 10 years after an ICU stay of at least 48 h. Williams reported that mortality in ICU survivors remained higher than the general population for every year during 15 years of follow up^3^. Although the precise effects are unknown thus far, it seems reasonable to argue that a continuously declining trajectory after ICU survival is also affecting HRQOL. Moreover, it is intriguing that survivors report comparable perceived quality of life (as judged by effect-size) when compared to baseline, despite potential major intercurrent health events. Of course, the data are reported on a group level, so in individual cases intercurrent events may have played a major role in their perceived quality of life. Overall the potential negative effects seem to tease out, since perceived quality of life domain scores do not show an import effect-size. Second, we only included patients on their first admission^41^, who also stayed in the ICU for more than 48 h. Therefore, the results may not be generalizable to the group of patients with a short ICU stay. However, in a recent study we showed that the group of patient who stayed shorter than 48 h in the ICU do not show a different HRQOL over time than those with a longer ICU stay^24^. Third, we chose to use proxies for pre-admission scores instead of a retrospective assessment by patients at ICU discharge^23^. This was done because the scores before treatment usually could only be scored retrospectively in the patients. Although this could have influenced the patient’s recollection of their previous health due to recall bias^55^, the use of proxies in this setting^27^ was validated in an earlier study by our group and by other studies^56,57^. Therefore, the results between proxy and patients measures should be interpreted with caution. Furthermore, the results may not be generalizable to other populations or staffing situations because this was a single center study. Information bias or selection bias could have played a role. However, we think that the latter factors do not play an important role in our study because all consecutive patients were eligible and actually evaluated and consistently only one researcher (JGMH) performed the quality of life evaluations. Finally, this study as in most long-term studies showed loss of follow up patients. This is a possible bias in the study, however we chose to analyze changes over time from ICU discharge using a linear mixed model. These models are less biased than complete-case analyses, as also the ‘worse’ patients who eventually drop out of the study are included as much as possible in the estimations of change over time. Including patients who dropped out during follow up will have had a negative impact on the estimates of improvement over time. In addition, we calculated the estimated marginal mean values of the physical component scores derived from the linear model.

## Conclusions

We showed that physical functioning of medical-surgical ICU survivors remains impaired at 10 years after ICU discharge compared with their pre-admission values and an age reference population. Effect sizes showed no significant differences compared with the pre-admission status suggesting that patients who survived largely regained their age-specific HRQOL at 10 years after ICU discharge. Further research on the identification and treatment of the new developed chronic conditions in long-term ICU survivors after 5–10 years seems essential if we are to optimize long-term outcomes after critical illness.

## Supplementary Information

Supplementary Information.

## Data Availability

All data generated or analysed during this study are included in this published article and its supplementary information files.

## Abbreviations

ICU: Intensive care unit
IQR: Interquartile range (P_25_-P_75_)
HRQOL: Health-related quality of life
SF-36: Short form-36
