# Supplementary material for: Health-related quality of life in ICU survivors—10 years later
Source: Sci Rep. 2021 Jul 26;11:15189. doi: 10.1038/s41598-021-94637-z (PMC8313552; doi:10.1038/s41598-021-94637-z)
Supplement: Supplementary file 1 — Supplementary Information. [file 41598_2021_94637_MOESM1_ESM.pdf]

Health-related quality of life in ICU survivors – 10 years later

José G.M. Hofhuis, RN, PhD[1], Augustinus J.P. Schrijvers PhD[3],

Tjard Schermer, PhD [2, 5] Peter E. Spronk, MD, PhD [1,4]

Department of Intensive Care[1] and Epidemiology[2] , Gelre Hospital,
Albert Schweitzerlaan 31, 7334 DZ Apeldoorn, The Netherlands
Julius Center for Health Sciences and Primary Care[3] , University Medical Center,
Heidelberglaan 100, 3584 CX Utrecht, The Netherlands
Department of Intensive Care, Academic Medical Center [4], Amsterdam, Meibergdreef 9,1105 AZ, Amsterdam, The Netherlands
Radboud Institute for Health Sciences, Radboud University Medical Center, [5] Geert Grooteplein 21, 6525 EZ, Nijmegen, the Netherlands

Additional file 1.

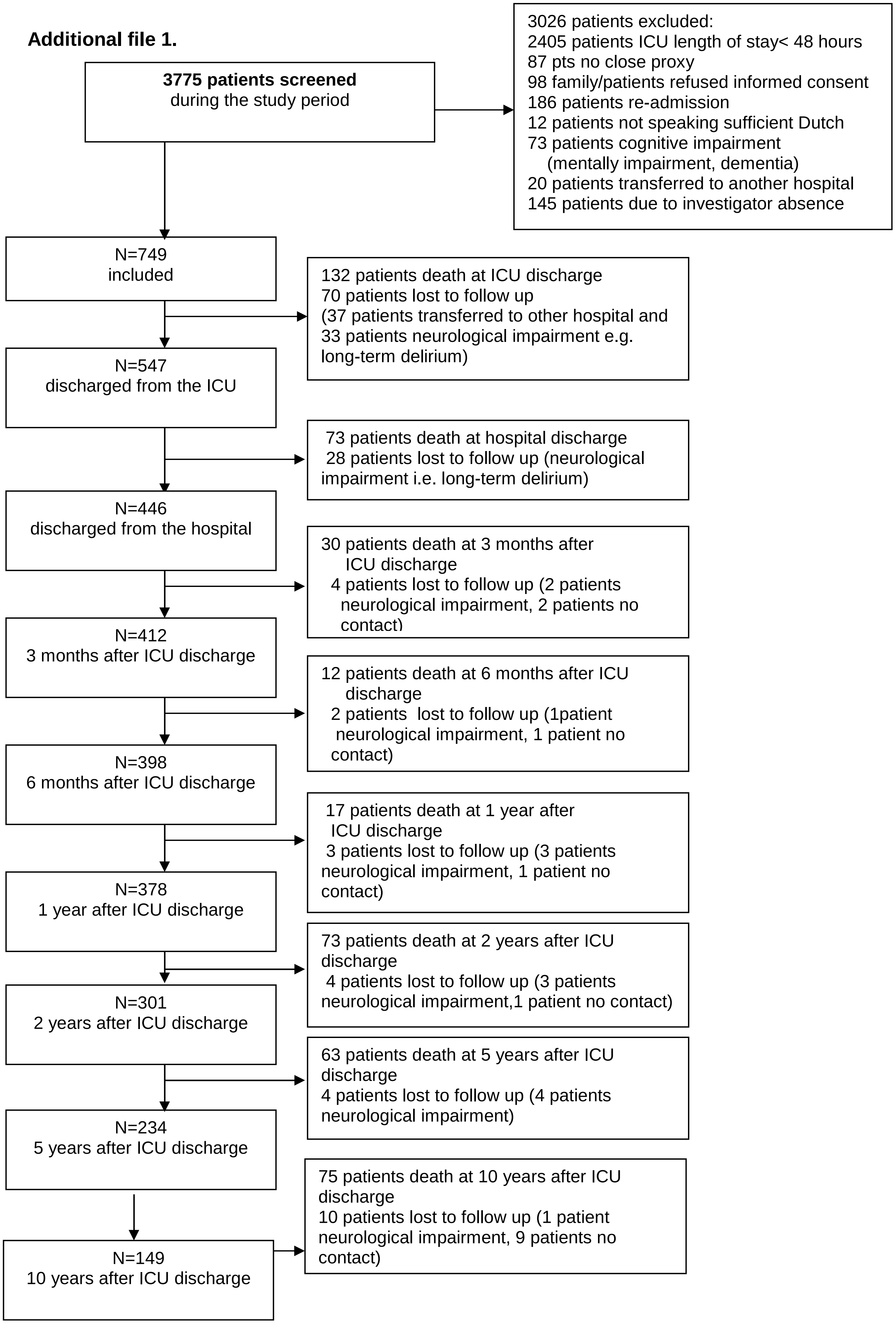

Additional file 2.

Demographic and clinical characteristics of analyzed group and lost to follow up

Median (IQR)	Group analyzed	Lost to follow up	P value
**N=**	624	125	
Age total group (years)	72 (63-78)	67 (50-75)	<0.001
**Sex: Male N (%)**	380 (60.9)	77 (61.6)	0.920
**Female N (%)**	244 (39.1)	48 (38.4)	0.920
Acute Physiology Age and Chronic Health Evaluation (points)	19 (15-23)	17 (12-22)	0.009
ICU length of stay (days)	8 (5-16)	7 (5-14)	0.661
Hospital length of stay (days)	23 (14-41)	20 (10-34)	0.053
Ventilation days	6 (3-12)	6 (3-11)	0.570
**Diagnostic groups N (%)**			
Cardiovascular pathology	156 (25)	28 (22.4)	0.571
Respiratory pathology	195 (31.3)	49 (39.2)	0.009
Gastrointestinal pathology	229 (36.7)	30 (24.0)	0.007
Neurological pathology	22 (3.5)	8 (6.4)	0.136
Trauma	14 (2.2)	9 (7.2)	0.007
Others	8 (1.3)	1 (0.8)	1.000
**Type of admission N (%)**			
Non-surgical	329 (52.7)	86 (68.8)	0.001
Emergency surgical	221 (35.4)	36 (28.8)	0.179
Elective surgical	74 (11.9)	3 (2.4)	0.001
**Type of proxy N (%)**			
Spouse	410 (65.7)	86 (68.8)	0.535
Children	206 (33)	33 (26.4)	0.172
Brother/Sister	9 (1.3)	6 (4.8)	0.026

Elective surgical: ICU admission was planned within a 24 hour period before surgery

Emergency surgical: unplanned surgery

Non-surgical: all other admissions

Values indicate medians and interquartile range (P_{25}-P_{75}) unless stated otherwise

Additional file 3.

Pre-admission Health-related quality of life of non-survivors compared with survivors

Short-Form-36 dimensions	Pre-ICU non-survivors 10 years	Pre-ICU Survivors 10 years	Differences survivors versus non-survivors 149/475		
N=	475	149	P value	Effect-sizes	95%CI
Physical component	38±13	48±11	<0.001	0.74	0.55-0.93
Mental component	48±11	52±9	<0.001	0.44	0.25-0.62
Physical functioning	79±26	52±34	<0.001	0.81	0.62-1.0
Role-physical	41±47	74±42	<0.001	0.71	0.52-0.90
Bodily pain	77±28	82±26	0.041	0.19	0.003-0.37
General health	46±28	66±28	<0.001	0.70	0.51-0.89
Vitality	49±23	66±25	<0.001	0.67	0.48-0.86
Social functioning	68±25	86±20	<0.001	0.71	0.53-0.90
Role-emotional	70±43	86±34	<0.001	0.39	0.20-0.57
Mental health	65±16	75±16	<0.001	0.63	0.45-0.82

Mean ± SD; 95%CI= Confidence interval.
